# Hardness of Polycrystalline Wurtzite Boron Nitride (wBN) Compacts

**DOI:** 10.1038/s41598-019-46709-4

**Published:** 2019-07-15

**Authors:** Yinjuan Liu, Guodong (David) Zhan, Qiang Wang, Duanwei He, Jiawei Zhang, Akun Liang, Timothy E. Moellendick, Le Zhao, Xiao Li

**Affiliations:** 1 Institute of Atomic and Molecular physics, Sichuan University, Chengdu, 610065 China; 2 Key Laboratory of High Energy Density Physics and Technology of Ministry of Education, Sichuan University, Chengdu, 610065 China; 3 Drilling Technology Division, Exploration and Petroleum Engineering Center - Advanced Research Center (EXPEC-ARC), Saudi Aramco, Dhahran, 31311 Saudi Arabia; 4 North China Institute of Aerospace Engineering, Langfang, 065000 China; 5 Weihai Weiying Tool Co., Ltd, Weihai, 264210 China

**Keywords:** Condensed-matter physics, Materials science

## Abstract

Wurtzite boron nitride (wBN), due to its superior properties and many potential practical and scientific applications, such as ideal machining/cutting/milling ferrous and carbide materials, especially as an ideal dielectric substrate material for optical, electronic, and 2-D graphene-based devices, has recently attracted much attention from both academic and industrial fields. Despite decades of research, there is an ongoing debate about if the single-phase wBN is harder than diamond because of the difficulty to make pure wBN material. Here we report the successful synthesis of pure single-phase polycrystalline wurtzite-type boron nitride (wBN) bulk material by using wBN powder as a starting material with a well-controlled process under ultra-high pressure and high temperature. The cubic boron nitride (cBN) was also successfully prepared for the first time from wBN starting material for comparison and verification. The X-ray diffraction (XRD) and TEM clearly confirmed that a pure single-phase wBN compact was produced. The microstructure and mechanical properties including Vickers hardness, fracture toughness, and thermal stability for the pure single-phase wBN was first evaluated.

## Introduction

Boron nitride is a kind of material which crystallizes in hexagonal, cubic and wurtzitic structures^1–3^. Hexagonal boron nitride (hBN) is a stable phase at ordinary temperature and pressure^4^, and cubic boron nitride (cBN) and wurtzitic boron nitride (wBN) can be synthesized at high temperature and high pressure^2,5–11^. With regard to the mechanical properties of cBN^12–16^, especially its hardness, about 45–50 GPa^17^, there is a certain amount of information that has been reported, however, almost nothing is known about the mechanical properties of wBN because wurtzite is a metastable phase of BN at all pressures and temperatures^18,19^ and it is difficult to prepare a pure phase^20^. Previous studies have shown that the hardness of wBN varies significantly from 24 GPa to 54 GPa^17,18^. Some studies suggested that wBN may be as hard as or even harder than diamond, which came as a surprise since wBN and cBN have a similar bond length, elastic moduli, ideal tensile and shear strength^20,21^. Therefore, it is very important to produce pure single-phase wBN bulk material to accurately measure its hardness.

In previous studies, the synthesis of wBN was directly transformed from hBN under high pressure and high temperature^10,18,22,23^. The other phases coexist in the transformed samples during the thermal and pressurized treatment or fabrication of sintered compacts^2,24^, and the resultant values cannot represent those of pure wBN. Here we for the first time applied pure wBN powder as starting material and successfully synthesized pure wBN and cBN compacts by means of the ultra-high temperature and high pressure under well controlled conditions. To clarify the long-standing debate in the academic community about whether pure wBN is harder than diamond or not, a comparative study of the mechanical properties, in particular, the hardness and fracture toughness of both pure wBN and cBN, together with their microstructures and thermal stability has been investigated in detail.

## Results

### Synthesis of pure wBN and cBN from wBN starting powders

Due to its stable structure at ordinary atmosphere, hexagonal boron nitride (hBN) powder has long been used as a starting material for the manufacturing of cBN and wBN. In our study, wBN powder was first explored as starting material for the synthesis of pure wBN and cBN bulk materials. Both pure wBN and cBN samples were obtained under ultra-high pressure and high temperature (UHPHT) conditions using a two-stage (6–8 system) large volume multi-anvil apparatus^25–27^. The recovered synthetic products were well-sintered bulk materials in cylindrical shape with a dimension of 2 mm in height and 2.5 mm in diameter that were polished to a mirror surface for further characterization.

**Figure 1:**
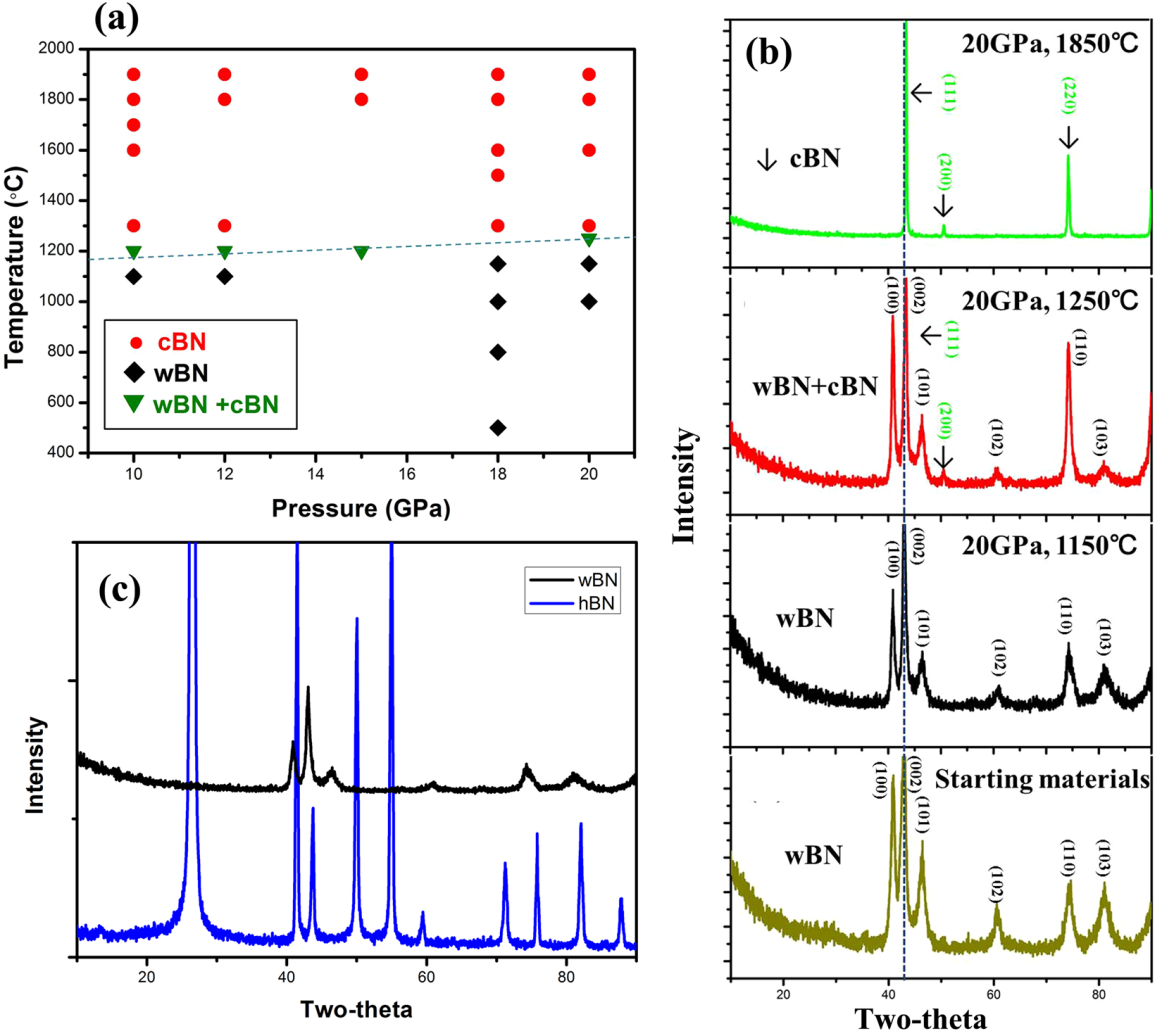
(**a**) Transformation pressure-temperature diagram of wBN (Red circle: cBN, Black rhombus: wBN, Green triangle: wBN + cBN). (**b**) XRD patterns of the starting wBN, and the synthesis products synthesized at 20 GPa and 1,150 °C, 1,250 °C and 1,850 °C, respectively. (**c**) XRD patterns of hBN and wBN.

We conducted a series of experiments under different sintering pressures and temperatures to study the wBN to cBN phase transformation P-T boundary (Fig. 1a). The phase purity of the synthesized samples was calibrated by the X-ray diffraction (XRD) analysis with CuKα radiation (DX-2700, Dandong, China). It is found that the UHPHT synthesized product from the wBN powder was either pure wBN or transformed pure cBN with a narrow mixture phase boundary. It is also very interesting to note that there is a narrow wBN to cBN transformation temperature window, irrespective to the pressures applied. No hBN was detected within the *P, T* conditions explored as shown in Fig. 1c. It is also very importantly noted that the sintering temperatures below 1,200 °C were necessary to remain pure wBN phase. Figure 1b shows XRD patterns of the starting wBN powder and the synthesized products at a constant pressure of 20 GPa under different sintering temperatures. At temperatures below 1,200 °C, no cBN phase from wBN is formed. Above 1,200 °C, very weak reflections of planes (111) and (200) of cBN are detected, suggesting the cBN formation temperature boundary and fully transformation process at 1,300 °C. The results show that the fully dense pure wBN and pure cBN compacts from wBN powders have been successfully synthesized under a pressure of 20 GPa at 1,150 °C and 1,850 °C, respectively, and the cooling process does not affect the properties of the sample due to the phase change has been completed at high temperatures and pressures.

**Figure 2:**
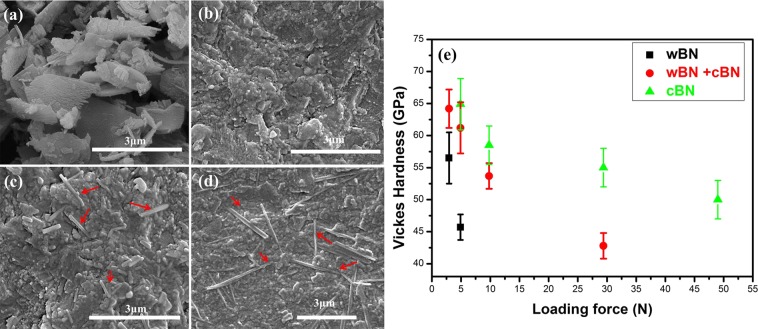
(**a**) SEM micrography of the initial wBN powder. (**b**) wBN compact SEM micrograph synthesized by 20 GPa and 1,150 °C. (**c**) wBN + cBN compact electron microscope photo synthesized by 20 GPa and 1,250 °C. (**d**) cBN compact SEM micrograph synthesized by 20 GPa and 1,850 °C. Insert: The enlarged SEM micrograph of (**d**). (**e**) In samples synthesized at 20 GPa and different synthesis temperatures of 1,150 °C, 1,250 °C and 1,850 °C, the hardness is changed as a function of the load force.

Microstructural analysis can be very intuitive to observe the microscopic morphology of the sample. To detect microstructure of the samples, the detailed microstructure of the synthesized and polished samples was investigated by Scanning Electron Microscopy (SEM) (JSM-6490, JEOL, Akishima, Japan). Figure 2a–d show the SEM micrographs of initial wBN powder, the synthesis of pure wBN, a mixture of wBN and cBN, and pure cBN compact, respectively. The initial wBN powder exhibits a lamellar microstructure, as shown in Fig. 2a. After a well-controlled HPHT treatment, we can obtain the completely dense pure wBN and cBN bulk materials. The wBN compact is augmented by ultra HPHT process with a layered or granular nano-sized microstructure while cBN compact consists of some lamellar structure in the nanostructure matrix having a grain size in the range of 50–200 nm. The results show that the slow heating speed (below 100 °C/min) is very important for obtaining good sintering specimens. As shown in Fig. 2c,d, the slow heating rate (100 °C/min) leads to the long-rod microstructure (microscopic structure marked with red arrows) because the wBN is converted to cBN. With the increase of heating rate, the grain size increases rapidly, the hardness decreases and the rod structure does not appear. In other words, the slow heating means that the heating rate cannot exceed 100 °C/min. When the heating rate is too fast the grain size increases rapidly, the hardness decreases and the rod structure does not appear”.

### Characterization

The hardness of polished specimens was tested with different loading forces and a fixed indentation time of 15 s by a Vickers hardness tester (FV-700, Japanese future technology). Under the action of 4.9N load force, the hardness of pure wBN, a mixture of wBN and cBN, and pure cBN compacts is measured. The average Vickers hardness value is determined according to the measurement results of five points on the specimen surface. The averages for measuring pure wBN, a mixture of wBN and cBN, and pure cBN compacts are: 46 ± 3 GPa, 61 ± 4 GPa, and 65 ± 5 GPa, respectively. The results show that the hardness of wBN increases with the increase of cBN content and proves that the hardness is lower than cBN. The results are effective because the hardness values of the cBN specimens are consistent with the previous research results^28–32^. In this paper, the hardness of the specimen is measured in detail, and the change of hardness as the loading force function is also studied, as shown in Fig. 2e. The results show that the hardness value of pure wBN is always lower than that of wBN-cBN and pure cBN compacts under different loads. Although it is reported that wBN may be more harder than diamond^21^, our findings suggest that the hardness of pure wBN is lower than that of pure cBN^17^ and diamond^33^. The value of Vickers hardness differs from the previously reported values because there are other phases and different loading forces^17,18,21^. No previous studies have shown that such a high-purity single phase for wBN. Our findings represent the true performance of pure wBN in bulk materials.

**Figure 3:**
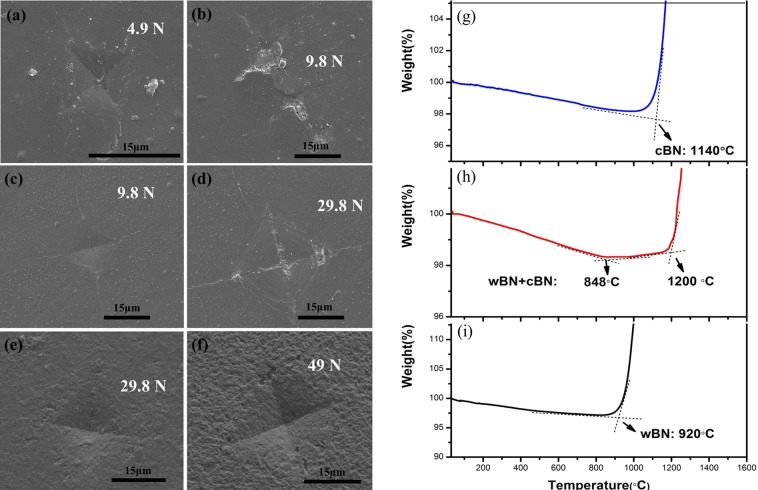
(**a,b**) Indentation of wBN compact at the loading force of 4.9N and 9.8N. (**c,d**) Indentation of wBN + cBN compact at the loading force of 9.8N and 29.4 N. (**e,f**) Indentation of cBN compact at the loading force of 29.4N and 49N. (**g**–**i**) TGA results of samples synthesized at 20 GPa and 1,850 °C, 1,250 °C and 1,150 °C, respectively.

Fracture toughness is a meaningful mechanical property of the sample. In order to compare the fracture toughness of wBN and cBN, the fracture toughness of wBN and cBN has been qualitatively evaluated through Vickers hardness indentation test. The scanning electron microscope images of Fig. 3 under different loading forces are pure wBN, a mixture of wBN and cBN, and pure cBN specimens, respectively. The results show that the fracture toughness of each specimen is strongly influenced by its phase composition. Under the load of 4.9N, pure wBN with compact indentation intact, when the loading force exceeds 9.8N, the pure wBN compact indentation almost cracked, as shown in Fig. 3a,b, respectively. With the appearance of pure cBN phase, the fracture toughness of the specimen increases, because the dents in Fig. 3c,d are visible, except for the cracks in the corners. Under a loading force of 4.9N, the indentation of pure wBN compact was intact, and when pure wBN is fully converted to pure cBN, even under the higher loading force of Fig. 3e,f, the indentation of the specimen does not appear to be cracked, suggesting that the fracture toughness of pure wBN is less than that of pure cBN compact.

Thermal Gravimetric Analysis (TGA) were measured to determine the oxidation resistance of samples and compared with cBN and Diamond. Thermal Gravimetric Analysis (TGA) (TG-Q600, TG-Q2000, USA) is carried out in air with a heating rate of 10 °C/minute from 30 °C to 1,400 °C in order to further investigate their thermal stability. Figure 3g–i show TGA results for pure wBN, a mixture of wBN and cBN, and pure cBN compacts, respectively. As can be seen, the pure wBN compact is thermally stable in air up to ~920 °C but it is lower than that of pure cBN compact (~1140 °C). This conclusion further illustrates the increased thermal stability in the mixture with increasing cBN content. This is the fact that when a part of the wBN is converted to cBN, there are two exothermic peaks in the thermal weight of the sample, i.e., the exothermic oxidation peaks of wBN and cBN. On the other hand, TGA shows that the thermal stability of pure wBN is much better than that of diamond (600 °C)^33^.

### Mechanism

**Figure 4:**
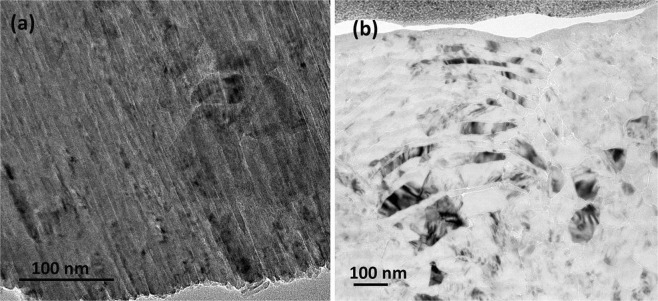
(**a**) TEM micrograph of initial wBN powder. (**b**) TEM micrograph of wBN compact synthesized at 20 GPa and 1,150 °C.

The transmission electron microscopy (TEM; JEM-2100F, JEOL, Japan) was used to investigate their detailed microstructures at higher magnifications. Figure 4 shows the TEM of starting material and specimen sintered at 20 GPa and 1,150 °C. The micrograph of initial wBN powder possesses a uniform strip microstructure (Fig. 4a). The results show that the microstructure of the specimen with ultra high temperature and high pressure is still banded, and there is no obvious change except the curvature of the strip shape (Fig. 4b). Combined with microscopic analysis of SEM, it is found that high pressure leads to strip structure bending in nanoscale. At the conversion temperature of wBN to cBN, the wBN morphology remains unchanged. This further confirms that the samples synthesized at 20 GPa and 1,150 °C are still pure wBN phases.

**Figure 5:**
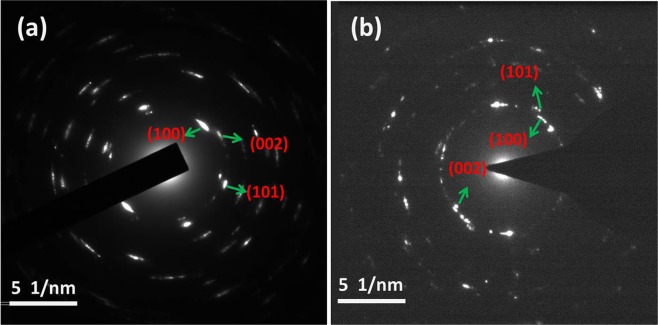
(**a**) Selected area electron diffraction pattern of initial wBN powder. (**b**) Selected area electron diffraction pattern of wBN compact synthesized at 20 GPa and 1,150 °C.

The corresponding selected area electron diffraction modes in Fig. 5a,b are the initial materials and samples synthesized by 20 GPa and 1,150 °C respectively. It shows that the main planes (100), (002) and (101) are present in the initial material (Fig. 5a) and the sample synthesized at 20 GPa and 1,150 °C (Fig. 5b), further confirming that the compact is pure wBN.

## Discussion

In summary, we have successfully synthesized pure polycrystalline wBN and cBN bulk materials directly from wBN starting powder by ultra HPHT technology for the first time. It is found that the usage of pure wBN initial material, the critical synthesis temperature control along with slower heating rates favor the synthesis of those pure BN compacts. Modern characterizations such as XRD, SEM, TEM, and SAD confirm the synthesis of pure single-phase wBN bulk materials. The Vickers hardness of pure polycrystalline wBN compact is first determined to be 46 GPa on average, not as thought as harder than diamond. The material exhibits a high thermal stability with an onset oxidation temperature at 920 °C in air that is much higher than diamond. The success of synthesis and performance evaluation of pure wBN bulk materials clarifies the long-standing debate if harder than diamond, which is important for both fundamental research as well as industrial tool and device applications.

## Methods

The wBN powders purchased from Weiying Superhard Materials Co., Ltd. were used as starting materials. In a vacuum furnace of 3.0 × 10^−3^ Pa, the powder was treated with 400 °C for one hour to remove the impurity gas attached to the grain surface. After vacuum heat treatment, the starting materials were contained in an Re capsule, and then were subjected to HPHT treatments under pressures of 10~20 GPa and temperatures of 400~1900 °C in a two-stage (6–8 system) large volume multi-anvil apparatus^25–27^. The pressure was calibrated by means of direct determination of known pressure-induced phase change and cell temperature with WRe3%–WRe25% thermocouple. Wurtzitic boron nitride powders have been compressed to pressure and heated with a 100 °C/minute heating rate to the desired temperature with a duration of 30 minutes. The sample was quenched to ambient temperature with a cooling rate of about 50 °C/min, and then decompressed to the surrounding pressure.
